# Circulating cell-free DNA-based methylation patterns for breast cancer diagnosis

**DOI:** 10.1038/s41523-021-00316-7

**Published:** 2021-08-16

**Authors:** Xianyu Zhang, Dezhi Zhao, Yanling Yin, Ting Yang, Zilong You, Dalin Li, Yanbo Chen, Yongdong Jiang, Shouping Xu, Jingshu Geng, Yashuang Zhao, Jun Wang, Hui Li, Jinsheng Tao, Shan Lei, Zeyu Jiang, Zhiwei Chen, Shihui Yu, Jian-Bing Fan, Da Pang

**Affiliations:** 1 Department of Breast Surgery, Harbin Medical University Cancer Hospital, Harbin, China; 2 Department of Research and Development, AnchorDx Medical Co., Ltd., Guangzhou, China; 3 Department of Pathology, Harbin Medical University Cancer Hospital, Harbin, China; 4 Department of Epidemiology, Harbin Medical University, Harbin, China; 5 AnchorDx, Inc., Fremont, California USA; 6 Guangzhou Kingmed Center for Clinical Laboratory Co., Ltd., Guangzhou, China; 7 Department of Pathology, School of Basic Medical Science, Southern Medical University, Guangzhou, China

**Keywords:** Breast cancer, Diagnostic markers

## Abstract

Mammography is used to detect breast cancer (BC), but its sensitivity is limited, especially for dense breasts. Circulating cell-free DNA (cfDNA) methylation tests is expected to compensate for the deficiency of mammography. We derived a specific panel of markers based on computational analysis of the DNA methylation profiles from The Cancer Genome Atlas (TCGA). Through training (*n* = 160) and validation set (*n* = 69), we developed a diagnostic prediction model with 26 markers, which yielded a sensitivity of 89.37% and a specificity of 100% for differentiating malignant disease from normal lesions [AUROC = 0.9816 (95% CI: 96.09-100%), and AUPRC = 0.9704 (95% CI: 94.54–99.46%)]. A simplified 4-marker model including *cg23035715*, *cg16304215*, *cg20072171*, and *cg21501525* had a similar diagnostic power [AUROC = 0.9796 (95% CI: 95.56–100%), and AUPRC = 0.9220 (95% CI: 91.02–94.37%)]. We found that a single cfDNA methylation marker, *cg23035715*, has a high diagnostic power [AUROC = 0.9395 (95% CI: 89.72–99.27%), and AUPRC = 0.9111 (95% CI: 88.45–93.76%)], with a sensitivity of 84.90% and a specificity of 93.88%. In an independent testing dataset (*n* = 104), the obtained diagnostic prediction model discriminated BC patients from normal controls with high accuracy [AUROC = 0.9449 (95% CI: 90.07–98.91%), and AUPRC = 0.8640 (95% CI: 82.82–89.98%)]. We compared the diagnostic power of cfDNA methylation and mammography. Our model yielded a sensitivity of 94.79% (95% CI: 78.72–97.87%) and a specificity of 98.70% (95% CI: 86.36–100%) for differentiating malignant disease from normal lesions [AUROC = 0.9815 (95% CI: 96.75–99.55%), and AUPRC = 0.9800 (95% CI: 96.6–99.4%)], with better diagnostic power and had better diagnostic power than that of using mammography [AUROC = 0.9315 (95% CI: 89.95–96.34%), and AUPRC = 0.9490 (95% CI: 91.7–98.1%)]. In addition, hypermethylation profiling provided insights into lymph node metastasis stratifications (*p* < 0.05). In conclusion, we developed and tested a cfDNA methylation model for BC diagnosis with better performance than mammography.

## Introduction

Early detection of breast cancer (BC) leads to a better prognosis. Although mammograms have been used in screening, their sensitivity varies from approximately 68 to 93%, depending on practitioner experience, patient age, breast density, and postmenopausal hormonal therapy, among other factors^1,2^. Therefore, there exists a need to develop accurate screening methods for BC.

BC-specific DNA methylation changes occur early during tumorigenesis^3,4^. Several studies have investigated the methylation status, efficacy and validity of using cell-free DNA (cfDNA) for cancer detection^5,6^, including the detection of BC^7,8^. A few clinical studies have since selected and elucidated candidate markers based on the beta-value of individual promoter CpG sites for early stage BCs^8–10^, but a single differentially methylated CpG island as a reliable and quantitative measurement of tumour burden in cfDNA has not been identified^11^. To enable genome-wide methylation profiling, we developed a highly sensitive, targeted DNA methylation sequencing technique for analysing the methylation status in cfDNA and named it the AnchorIRIS^TM^ assay^12^.

We first derived a specific panel of markers based on computational analysis of the DNA methylation profiles from The Cancer Genome Atlas (TCGA). After experimental evaluation of the panel markers in matched tumour genomic DNA and plasma cfDNA, a diagnostic model was developed and further tested in an independent group. Overall, the analyses showed that cfDNA methylation profiling may serve as a reliable approach for BC diagnosis. With only a few markers, the model can be widely applied to large-scale BC screening at a low cost.

## Results

### Methylation panel development in BC

**Fig. 1:**
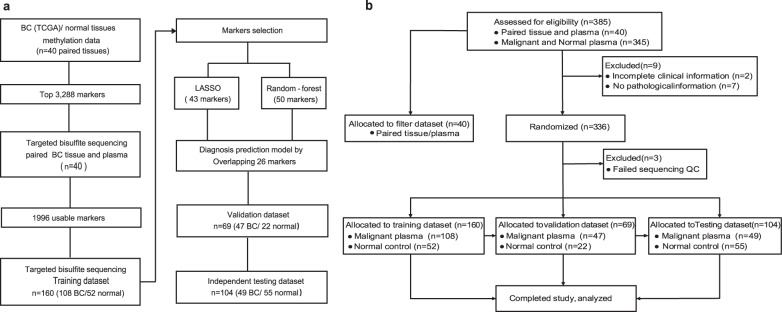
Workflow of the study design and Consort diagram. **a** The breast cancer (BC) methylation panel targets were specifically selected as those areas with differences in methylation between tumour and normal tissue based on The Cancer Genome Atlas (TCGA) data. Forty paired tissue and plasma samples were used to identify a total of 1996 methylation pattern markers. Targeted methylation profiling for cancer/normal classification was performed as follows: LASSO and random forest analyses were applied to a training cohort of 52 normal controls and 108 breast cancer patients to identify a final selection of 26 markers. These 26 markers were applied to a validation cohort of 22 normal controls and 47 breast cancer patients. The final performance of our models was tested in an independent test cohort of 55 normal controls and 49 breast cancer patients. **b** Participants screened and enrolled. QC quality criteria. Samples in tissue and plasma underwent targeted capture-probe sequencing via high-throughput sequencing with the ultrasensitive AnchorIRIS^TM^ assay. Our goal was to select enough controls and malignant plasma, but due to the available clinical information of samples and exclusion criteria, we were not able to identify all assessed samples that entered the analysis. Finally, 40 pairs of breast cancer tissue DNA and matched plasma cfDNA were used for filtering, and 336 plasma cfDNA were randomly assigned to a training group (*n* = 160), validation group (*n* = 69) and independent testing group (*n* = 104).

Figure 1a, b and Table 1 show the study design, flowchart of the enrolled participants and the qualified plasma used for constructing diagnostic model, respectively. We hypothesised that CpG markers with a maximal difference in methylation between tumour and normal tissues would be most likely to differentiate BC patients from normal controls in terms of cfDNA methylation profiles. The “Wilcoxon rank-sum test” was applied to discover differentially methylated loci. We set the false discovery rate (FDR) significance level at 0.05 and the least difference of mean beta value between tumour and normal tissues at 0.2 and developed a specific methylation panel targeting approximately 3288 CpG sites by analysing 40 paired BC tumour and normal samples from TCGA (Fig. 1a). Unsupervised hierarchical clustering of these markers could differentiate between tumour and normal samples (Supplementary Fig. 1a). Analysis of the genomic distribution showed that 50.43% of the markers were located in CpG islands in proximal promoters (within 1500 bp upstream of the transcription start sites and in 5′UTRs), 1.67% were located in 3′UTRs, 29.65% were located in gene bodies, and 29.68% were in intergenic regions (Supplementary Fig. 1b).

**Table 1:** Characteristics of healthy volunteers and patients with breast cancer.

Characteristics	Subgroup	Whole set (*n* = 333)	Training set (*n* = 160)	Validation set (*n* = 69)	Testing set (*n* = 104)
Number of samples	Healthy control	129	52	22	55
	Patients with BC	204	108	47	49
Age (patients/healthy)	<40	3/6	2/0	1/0	0/6
	40–49	88/59	51/30	22/11	15/18
	50–59	85/50	47/21	19/8	19/21
	≥60	28/14	8/1	5/3	15/10
	Mean	51.88/49.4	50.4/49.2	51.4/49.7	55.6/49.3
Subtype	Luminal A	53	30	12	11
	Luminal B (HER2+)	31	12	7	12
	Luminal B (HER2−)	58	32	16	10
	HER2	15	8	4	3
	TNBC	23	13	4	6
	NA (DCIS)	24	13	4	7
Stage	0	11	5	3	3
	I	68	40	12	16
	II	91	44	22	25
	III	32	19	8	5

*NA* not assessed, *DCIS* ductal carcinoma in situ, *BC* breast cancer.

With the ultrasensitive AnchorIRIS^TM^ assay, we can measure the methylation status of lower-input cfDNA while maintaining sufficient diversity and sensitivity^12^. The 3288 markers were tested in 40 pairs of BC tissue DNA and matched plasma cfDNA. A total of 1996 co-methylated region markers with dynamic methylation ranges were selected using the ‘moderated *t*-statistics’ method with empirical Bayes to shrink the variance and the Benjamini–Hochberg procedure to control the FDR between the tissue and plasma samples (FDR < 0.05). The methylation profiles in BC tumour DNA and matched plasma cfDNA were consistent (Fig. 1a and Supplementary Fig. 2). Through filtering the noise methylation patterns between the matched tissues and plasma, 1996 markers were selected for further analysis (Supplementary Fig. 3).

### Train and validation of the methylation panel in BC diagnosis

**Fig. 2:**
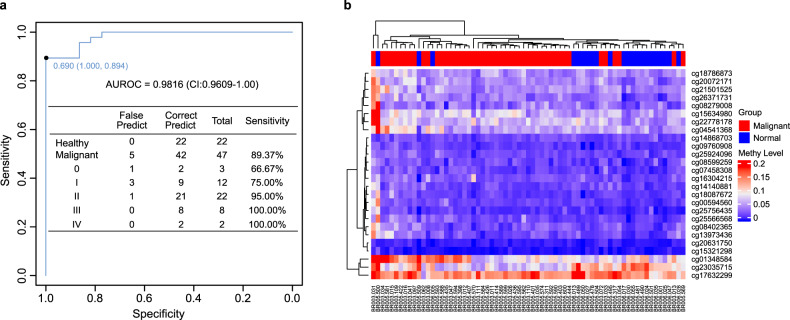
Performance of the diagnostic model in a validation plasma cohort. **a** The validation group was composed of 47 breast cancer and 22 normal plasma samples. ROC curve for breast cancer detection using the final model with 26 markers. This model achieved the best diagnostic power with an AUROC of 0.9816 (95% CI: 96.09–100%) in the validation dataset. **b** Heatmap for cfDNA methylation levels of the validation group. Cancer or normal samples could be distinguished through methylation patterns in the validation dataset.

To select markers and construct a diagnostic model from the cfDNA samples, we modelled the 1996 markers in the training cohort (108 BC and 52 normal cfDNA samples) by random forest and LASSO methods. We obtained 50 markers and 43 markers by random forest and LASSO analyses, respectively, in which 26 markers were overlapped (Fig. 1a and Supplementary Table 1). These 26 markers were applied to construct a diagnostic prediction model for BC with a sensitivity of 87.96% (95% CI: 82.57–94.5%) and specificity of 98.07% (95% CI: 94.12–100%) in the training cohort (Supplementary Fig. 4a). In the validation cohort, which was composed of 47 BC and 22 normal cfDNA samples, the sensitivity was 89.37% (95% CI: 78.72–97.87%), and the specificity was 100% (95% CI: 86.36–100%) (Fig. 2). For stages 0–III the model yielded a sensitivity of 60.00% (3/5), 80.00% (32/40), 95.45% (42/44), and 94.74% (18/19), respectively, and a specificity of 98.07% (51/52) in the training dataset (Supplementary Fig. 4a). In the validation dataset, this model yielded a sensitivity of 66.67% (2/3), 75.00% (9/12), 95.00% (21/22), 100% (8/8), and 100% (2/2) for stages 0–IV, respectively, and a specificity of 100% (22/22) (Fig. 2a). We demonstrated that this model could differentiate BCs from normal controls in both the training dataset with AUROC = 0.9839 (95% CI: 97–99.78%), AUPRC = 0.956 (95% CI: 94.21–97%) (Supplementary Fig. 4a, b) and the validation dataset with AUROC = 0.9816 (95% CI: 96.09–100%) (Fig. 2a). Unsupervised hierarchical clustering of these 26 markers could distinguish BCs from normal controls with high specificity and sensitivity (Fig. 2b and Supplementary Fig. 4c).

**Fig. 3:**
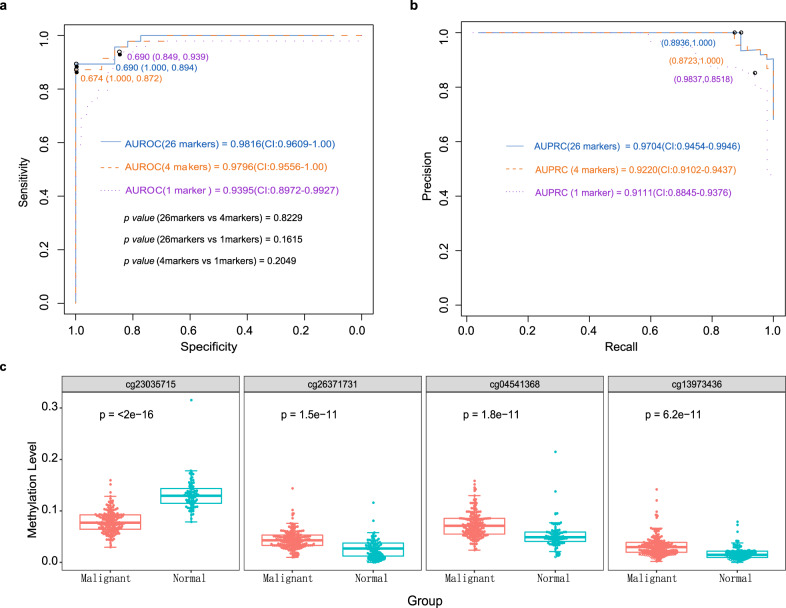
ROC and PRC curves using different models and methylation levels of four markers between malignant and normal plasma samples. **a**, **b** In the validation group, three different models were used for cancer detection. The first model included 26 markers (Supplementary Table 1) and achieved the best diagnostic power, with AUROC of 0.9816 (95% CI: 96.09–100%), and AUPRC of 0.9704 (95% CI: 94.54–99.46%). The second model included 4 markers (Supplementary Table 1) and achieved similar diagnostic power, with AUROC of 0.9796 (95% CI: 95.56–100%), and AUPRC of 0.9220 (95% CI: 91.02–94.37%). This model is suitable for PCR-based methods in future cancer screens. The last model included 1 marker, *cg23035715*, which achieved the best diagnostic power based on a single marker, with AUROC of 0.9395 (95% CI: 89.72–99.27%), and AUPRC of 0.9111 (95% CI: 88.45–93.76%). **c** Box plot showing the top four markers of increased and decreased methylation levels in malignant (*n* = 155) vs. normal (*n* = 74) plasma samples. The methylation level of *cg23035715* in normal plasma is higher than that in breast cancer plasma. It is different from other markers. *P* values were calculated using analysis of variance. For each box plot, the centre line, the boundaries of the box, the ends of the whiskers and points beyond the whiskers represent the median value, the interquartile range, the minimum and maximum values, and the outliers, respectively.

To confirm the contributions of each individual marker in the diagnostic model, we analysed 26 markers with single-factor logistic regression (Supplementary Table 1). Thus, we further adjusted the model to have as few markers as possible while maintaining similar diagnostic power with a simplified model using the top 4 markers (each marker has an AUROC above 0.75, resulting in an overall AUROC = 0.9796 (95% CI: 95.56–100%), AUPRC = 0.9220 (95% CI: 91.02–94.37%). Remarkably, *cg23035715* had the best diagnostic power, with an AUROC of 0.9395 (95% CI: 89.72–99.27%) and AUPRC of 0.9111 (95% CI: 88.45–93.76%) (Fig. 3a, b). This targeted region is hypomethylated in BC patients, while the other three sites are hypermethylated (Fig. 3c and Supplementary Table 1).

### Test of candidate DNA methylation markers with an independent cohort

To evaluate the universality of candidate markers, we analysed the methylation data of an independent test cohort, including 49 malignant and 55 normal donor plasma samples.

**Fig. 4:**
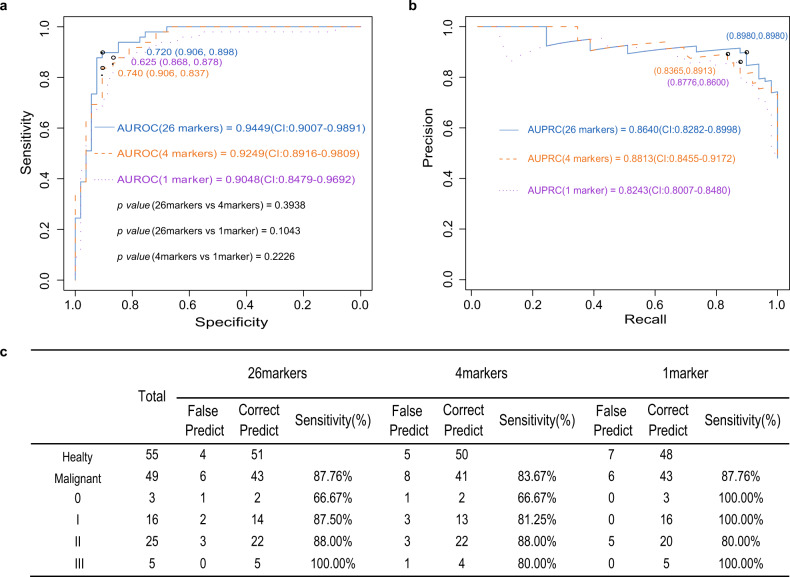
cfDNA methylation analysis for BC diagnosis in an independent testing cohort. **a**, **b** Receiver operating characteristic (ROC) curves and the precision–recall curve (PRC) with associated areas under the curves (AUCs) of the diagnostic prediction model using methylation analysis of different combined markers (top 1, 4, and 26 markers) in the independent testing cohorts. **c** For different stages, the top 1, 4, and 26 markers model yielded relatively consistent sensitivity.

Then, we showed the diagnostic performance of 1, 4, and 26 markers with receiver operating characteristic (ROC) curves and precision–recall curve (PRC), and the associated areas under the curve (AUCs) of 0.9048 (95% CI: 84.79–96.92%) vs. 0.8243 (95% CI: 80.07–84.80%), 0.9249 (95% CI: 89.16–98.09%) vs. 0.8813 (95% CI: 84.55–91.72%) and 0.9449 (95% CI: 90.07–98.91%) vs. 0.8640 (95% CI: 82.82–89.98%), respectively (Fig. 4a, b). The obtained diagnostic prediction model demonstrated a sensitivity of 87.76% (95% CI: 77.55–95.92%) and a specificity of 92.73% (95% CI: 84.91–98.11%) for discrimination of BR patients from normal controls in an independent testing dataset (*n* = 104) (Fig. 4c). These results demonstrated that cfDNA methylation analysis may contribute to BC diagnosis. However, this application needs further large-scale methylation-based prospective investigation in a BC population with longer clinical follow-up.

### Diagnostic power comparison of methylation markers and mammography

**Fig. 5:**
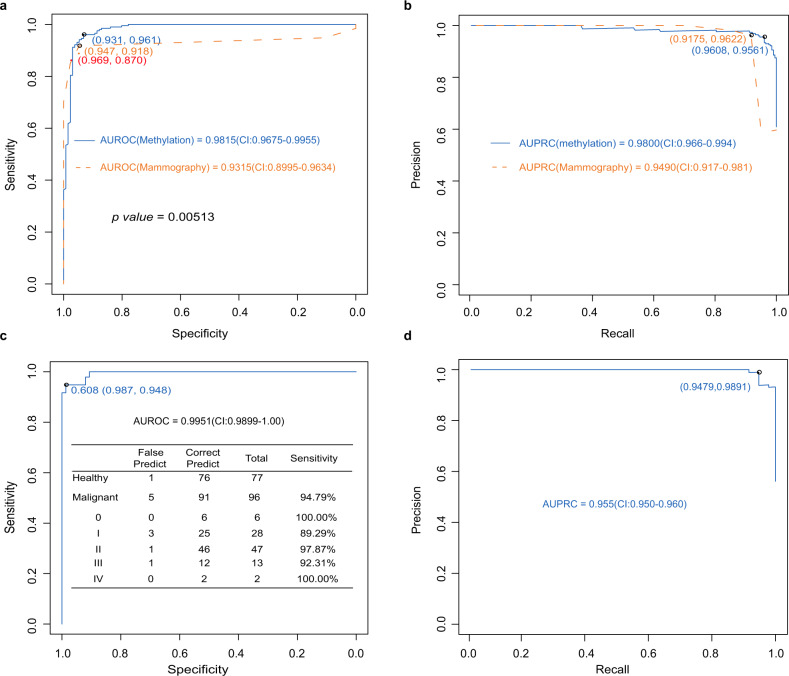
Diagnostic power comparison of methylation markers and mammography. **a, b** The methylation panel has better diagnostic power [AUROC = 0.9815 (95% CI: 96.75–99.55%), and AUPRC = 0.9800 (95% CI: 96.6–99.4%)] than mammography [AUROC = 0.9315 (95% CI: 89.95–96.34%), and AUPRC = 0.9490 (95% CI: 91.7–98.1%)]. **c**, **d** The methylation markers and mammography combined model has obvious advantages in specificity of 98.67% and sensitivity for different stage with AUROC of 0.9951 (95% CI: 98.99–100%) and AUPRC of 0.955 (95% CI: 95.0–96.0%).

We compared the diagnostic power of our 26 methylation markers panel with mammography in all participants (*n* = 333). The methylation panel has better diagnostic power [AUROC = 0.9815 (95% CI: 96.75–99.55%), and AUPRC = 0.9800 (95% CI: 96.6–99.4%)] than that of mammography [AUROC = 0.9315 (95% CI: 89.95–96.34%), and AUPRC = 0.9490 (95% CI: 91.7–98.1%)] for BC diagnosis in our cohort (*P* = 0.00513, Cohen’s *d* = 2.8127, Fig. 5a, b).

These 26 markers and mammography were applied to construct a combined diagnostic prediction model for BC. In the validation and the independent test cohort, which was composed of 96 BC and 77 normal cfDNA samples, the sensitivity was 94.79% (95% CI: 78.72–97.87%), and the specificity was 98.70% (95% CI: 86.36–100%) (Fig. 5c). For stages 0–IV the model yielded a sensitivity of 100% (6/6), 89.29% (25/28), 97.87% (46/47), 92.31% (12/13) and 100% (2/2), respectively (Fig. 5c). We demonstrated that this combined model could differentiate BCs from normal controls with an outstanding performance [AUROC = 0.9951 (95% CI: 98.99–100%) and AUPRC = 0.9550 (95% CI: 95.0–96.0%)] (Fig. 5c, d). The sensitivity relatively increased by 6.25% for the combined model (94.79%) compared with the methylation model (88.54%). The specificity increased by 3.89% for the combined model (98.70%) compared with the methylation model (94.81%).

### Correlation between clinicopathologic features and methylation levels

We investigated the association between clinicopathological features and methylation levels in 204 plasma samples of BC, including the training dataset, validation dataset and independent testing dataset (Supplementary Tables 2 and 3). Methylation levels were calculated in each individual in all subgroups (Supplementary Table 4). We performed ANOVA and Bonferroni correction to compare the means between clinicopathologic features and found that DNA hypermethylation was significantly associated with tumour size (*P* = 5.9E−08, *P*_adjusts_ = 1.12E−06), the number of metastatic lymph nodes (*P* = 1.2E−05, *P*_adjusts_ = 2.3E−04), stage (*P* = 7.3E−05, *P*_adjusts_ = 1.39E−03) and BC subtype (*P* = 0.029, *P*_adjusts_ = 0.55). Furthermore, no statistically significant associations were found for cfDNA hypermethylation with other parameters, including patient age (*P* = 0.18), grade (*P* = 0.91), ER status (*P* = 0.12), PR (*P* = 0.16) and HER2 status (*P* = 0.61). There were no significant associations for DNA hypomethylation with any clinicopathological parameter (Supplementary Table 2). In addition, according to the validation dataset and independent testing dataset, a box plot comparing the clinicopathological parameters according to hypermethylation levels (Supplementary Fig. 5a–f) and hypomethylation levels (Supplementary Fig. 6a–f) is illustrated. There were no significant associations between mean methylation levels in different clinicopathological features except the number of metastatic lymph nodes with mean hypermethylation level (Supplementary Fig. 5d).

## Discussion

Mammography-based screening has contributed to a 28–45% reduction in BC mortality^13,14^, showing 70% sensitivity and 92% specificity for BC detection^15^. However, mammography is less sensitive in young women, especially in Asian women, who usually have dense breast tissue. We need novel and accurate detection methods to complement the deficiencies of mammography in early BC detection.

Many studies have demonstrated the utility of cfDNA-based methylation biomarkers for the molecular characterisation of cancer and potential applications in diagnosis and prognosis^11,16^. The majority of cfDNA is debris from blood cells. Generally, 3–10 ng cfDNA can be extracted per millilitre of plasma (3–6 mL plasma out of every 10 mL blood) in healthy individuals^17^. In cancer patients, tumour-related cfDNA comprises 0.01–50% of plasma cfDNA^18^, which means that high-efficiency library construction and sensitive detection of cfDNA are necessary and challenging for early cancer diagnosis. The AnchorIRIS^TM^ assay demonstrated a very low limit of detection and limited amounts of input cfDNA (ranging from 1 to 10 ng)^12^, facilitating the use of cfDNA as a diagnostic biomarker from a typical 10 mL blood draw.

Our results demonstrated that the methylation patterns in plasma cfDNA samples can accurately predict the presence of BC. The sensitivity of detection in patients with stage 0–IV BCs were 66.67% (2/3), 75.00% (9/12), 95.00% (21/22), 100% (8/8) and 100% (2/2) respectively, in the validation set, with an AUROC of 0.9816 (95% CI: 96.09–100%). Previous studies conducted in the USA, Europe and Asia indicated that the overall sensitivity of mammography ranges from 74.6 to 92.5% and that the specificity ranges from 83.1 to 99.5%^2,19^. Specifically, the peak age for BC patients is between 40 and 60 years in Asian countries. Furthermore, approximately 49.2% of Asian women were categorised as having dense breasts (BI-RADS density 3 or 4), and 50.8% were fatty breasts (BI-RADS density 1 or 2)^1^. In Asian women, the sensitivity and specificity of BC screening using mammography are compromised. Considering that the major patients enrolled in our study were 40–60 years old, the detection model was more efficient and convenient than mammography. However, we also demonstrated that the methylation and mammography combined model could differentiate BCs from normal controls with an outstanding performance AUROC = 0.9951 (95% CI: 98.99–100%) and AUPRC = 0.955 (95% CI: 95.0–96.0%) (Fig. 5c, d). These results suggest that liquid biopsy is a promising non-invasive method for early cancer detection.

Our study also attests to the feasibility of ctDNA methylation analysis for patients with early stage BC. The classifier achieved consistently high and consistent sensitivity between cross-validation training and validation set [60.00% (3/5) vs. 66.67% (2/3)] in stage 0, [80.00% (32/40) vs. 75.00% (9/12)] in stage I, respectively. In the independent test set, sensitivity was 66.67% (2/3) in stage 0 and 87.5% (14/16) in stage I. Stage zero (stage 0) BC is also known as carcinoma in situ. According to the American Cancer Society, people with a type of BC that has not spread beyond the breast tissue have a 5-year survival rate of 99%. In addition, in stage I, the tumours are small and have spread very little, if at all. Our results show good performance in the diagnosis of carcinoma in situ and was better than the performance in stage I–II from Grail’s study^20^. Sensitivity increased with increasing stage of disease with our results, showing aberrant methylation occurring in developmental stages.

Using targeted bisulfite sequencing of cfDNA, we identified dozens of CpG markers with highly differential methylation patterns between BC and normal plasma samples. Cg23035715 (*TLR5*) has the best diagnostic power and is the main contributor to the 26-marker and 4-marker models. Its methylation level is lower in BC (hypomethylation), while most of the other markers are hypermethylated. Zhang *et al*. previously reported that *TLR5* is highly expressed in BC and that the TLR5 signalling pathway is overly responsive in BC cells^21^. Our results indicate that *TLR5* gene expression in BC may be due to intron hypomethylation, although further studies are needed to confirm this relationship. Other markers, such as *OTP*, *FEZF2* and *TSHZ3*, are involved in the tumorigenesis of BC and nasopharyngeal carcinoma^22–24^. The high cost is a major obstacle to implementing NGS in cancer screening. Therefore, we attempted to reduce our marker number in the model with the hope of converting it into PCR-based tests. We chose the top 4 of our 26 markers (each had an AUROC above 0.75) to construct the new model. The resulting model had similar diagnostic power to the 26-marker model (Fig. 3). We will test and validate this model in a large prospective cohort, with the aim of developing a cost-effective and user-friendly test for BC screening.

In addition, the diagnostic performance of plasma methylation analysis, which effectively distinguished BC patients with significantly different stages, was validated in an independent test cohort, including 49 malignant and 55 healthy controls. We showed that 26 marker tests identified 43 of 49 participants with BC, with a sensitivity of 87.76% (95% CI: 77.55–95.92%), and a specificity of 92.73% (95% CI: 84.91–98.11%). The sensitivity of detection in patients with stage 0–III BC were 66.67% (2/3), 87.50% (14/16), 88% (22/25) and 100% (5/5), respectively, with an AUROC of 0.9449 (95% CI: 90.07–98.91%) and AUPRC of 0.8640 (95% CI: 82.82–89.98%). Our results indicated that methylation analysis was superior to other currently reported cfDNA methylation markers for BC diagnosis and classical mammography tests. The diagnostic model described here provides a non-invasive, effective screening tool with likely good compliance for the early detection of BC.

Although several studies have elucidated the molecular subtypes of BC associated with gene expression patterns and specific methylation profiles^25,26^, our results did not find a direct correlation between methylation patterns and subtypes or histological grades. We found that tumour size, lymph node status, stage and subtype were associated with cfDNA hypermethylation (Supplementary Table 2). Tumour size and lymph node status are the main components of the TNM stage and are associated with methylation profiles^27^. Therefore, it is not surprising that methylation levels increase with stage progression. These results suggest that cfDNA methylation levels are a good indicator of tumour burden, which provides an excellent venue to non-invasively monitor tumour size, lymph node status and stage and a dynamic, series measurement to assess the overall prognosis. In BC, there is an epigenome-wide hypomethylation level of blood DNA^28^, and our results showed that the hypomethylation of *cg23035715* and *cg15321298* was significantly associated with PR status (*P* = 0.026, supplementary Table 2). Although the mechanism by which *cg23035715* and *cg15321298* hypomethylation affects PR status remains unclear, we suggest that hypomethylation might represent the effect of endocrine therapy as a surrogate marker.

Our study has several limitations. The study included possible bias due to the limited number of patients enrolled, which might hamper the statistical power. In addition, our training and validation cohorts included heterogeneous populations, including a limited age range of 40–60 years, various histology types and stages from 0 to III. Although we constructed a 4-marker model with similar diagnostic power, compared to the 26-marker model, which is a powerful model for BC screening, we need another screening cohort with a large sample size to determine the clinical utility of early detection in BC.

In summary, we developed a diagnostic model based on cfDNA methylation patterns, which serves as a reliable approach for the early diagnosis of BC. The simplified four-marker model holds great clinical potential for early BC diagnosis and screening.

## Methods

### Patients and sample collection

Participants included 204 female patients 37–76 years old, with an average age of 51 years and with histologically confirmed BC. Among these patients, 108 were in the training group, 47 were in the validation group, and 49 were in an independent testing dataset. A total of 129 healthy adult volunteers without BC participated in the study. Among these volunteers, 52 were in the training group, 22 were in the validation group, and 55 were in the independent testing group. The age distribution of these healthy donors was 40–60 years, with an average age of 49 years. Ten millilitres of blood was drawn 1–3 days prior to surgery and stored in Cell-Free DNA BCT^®^ blood collection tubes (Streck BCTs, Streck, Omaha NE. Cat# 218962) at room temperature. When the samples arrived at the laboratory, we centrifuged the tubes at 1,600×g for 10 min, removed the upper plasma layer and transferred it to a new conical tube, and then centrifuged the plasma at 16,000×*g* for 10 min. Plasma samples were collected and stored at −80 °C. Enrolled samples included 40 paired tissue-plasma samples that were used to evaluate the methylation concordance between tissue and plasma within the same individual.

All plasma and tissue samples were obtained from the Department of Breast Surgery, Harbin Medical University Cancer Hospital.

### Data pre-processing and marker selection

CpG sites with differential methylation levels between tumour and normal tissues in TCGA data were specifically selected as BC panel targets. A total of 3288 CpG sites were selected. Probe sequences for targeted CpG sites were selected from the Infinium HM450 array (Illumina, San Diego, CA). Enrichment probes were designed based on the Infinium Human Methylation 450 Bead Chip as 60 mer liquid hybridisation probes that could capture both the methylated and unmethylated forms of bisulfite-converted DNA after amplification. Probes were individually synthesised and 5′-biotinylated at TWIST BIOSCIENCE.

### DNA extraction of tissue and plasma

Tissue genomic DNA (gDNA) was isolated from fresh frozen tissue samples using a Qiagen QIAamp DNA Tissue Kit (Qiagen, Cat# 56404) according to the manufacturer’s protocol and was quantified using the NanoDrop^™^ and Qubit^™^ systems. gDNA was fragmented to 200 bp using an M220 Focused-ultrasonicator^™^ (Covaris, Inc.) The size was assessed by agarose gel electrophoresis. Fifty nanograms of fragmented DNA was used for library construction. Blood was collected in Streck BCT and transported within 4 days at room temperature. Repeated freezing and thawing of plasma were avoided to prevent cfDNA degradation and gDNA contamination from white blood cells (WBCs). cfDNA was isolated using a MagMAX^™^ Cell-Free DNA Isolation Kit (Thermo Fisher, Cat# A29319) according to the manufacturer’s protocol. The concentration of cfDNA was measured using a Qubit^™^ dsDNA HS Assay Kit (Thermo Fisher Scientific, Cat# Q32854), and quality was examined using an Agilent High Sensitivity DNA Kit (Cat# 5067-4626). cfDNA with a yield greater than 3 ng without excessive genomic DNA contamination was subjected to library construction.

### AnchorIRIS^™^ assay library preparation and sequencing

Extracted cfDNA was bisulfite-treated and purified using an EZ DNA Methylation-Lightning Kit (Cat# D5031, Zymo Research) according to the manufacturer’s protocol. Whole-genome or cfDNA amplification of bisulfite-converted DNA was performed using an AnchorDx EpiVisio^TM^ Methylation Library Prep Kit (AnchorDx, Cat# A0UX00019) and an AnchorDx EpiVisio^TM^ Indexing PCR Kit (AnchorDx, Cat# A2DX00025) following recommended conditions. Target enrichment was performed using an AnchorDx EpiVisio^TM^ Target Enrichment Kit (AnchorDx, Cat# A0UX00031) to specifically pull-down DNA fragments that contained target CpG sites using 5′-biotinylated capture probes. A total of 1000 ng DNA containing up to four pre-libraries was pooled for target enrichment using an AnchorDx PanMet V2-Pan-cancer methylation panel (AnchorDx, Cat# A0UX00023). Target capture libraries were sequenced on an Illumina NovaSeq 6000 Sequencer using 300 cycle runs. A 25% PhiX solution was spiked into the bisulfite sequencing libraries to increase base diversity for better sequencing quality.

We performed deep sequencing of the targeted bisulfite libraries to achieve 2000× mean target coverage (approximately 600 mean deduped coverage). FASTQ files were generated using Trim Galore version 0.4.1 (https://github.com/FelixKrueger/TrimGalore) and mapped to the bisulfite-converted hg19 reference genome using Bismark version 0.15.0 (Bowtie2 is the default aligner behind Bismark). Reads containing more than one alignment location, and PCR duplicates flagged by Picard (v1.129) were removed from further downstream analyses. On average, 85% of reads were properly aligned, and 65% were on-target. The assay showed a high uniformity of 97% (defined as the percentage of sites covered at a 0.2 mean depth), with only 0.06% of target sites having zero coverage.

### Predictive modelling of a malignant or normal state

We designed probes corresponding to the 3288 markers and tested them in 40 pairs of BC tissue DNA and matched plasma cfDNA from the same patient. Differentially methylated CpGs were identified by comparing BC to normal samples (FDR < 0.05, delta > 0.5) and further assembled into differentially methylated regions. Reads having at least 3 methylated CpGs within a sliding window of 3–5 CpGs were designated as co-methylated reads and used for subsequent analysis of methylation level (the percentage of co-methylated reads: co-methylated reads/all mapped reads with at least 3 CpGs) and predictive modelling of the malignant/normal states of patient samples. The methylation profiles in BC tumour DNA and matched plasma cfDNA were consistent (Supplementary Fig. 2). A total of 1996 markers with a good experimental amplification profile and dynamic methylation range were selected for further analysis.

To validate the collective prediction power of candidate markers, we used random forest and least absolute shrinkage and selection operator (LASSO) models in the training cohort of BC patients. We repeated 2-fold cross-validation 20 times and selected the top 50 markers by their importance scores in the random forest model. We also obtained 43 markers using a LASSO analysis in which we required selected markers to appear over 300 times out of a total of 500 repetitions. There were twenty-six overlapping markers between these 2 methods (Fig. 1). Using the random forest method, we constructed a diagnostic prediction model with these 26 markers that preferentially discriminated malignant samples from normal samples in the independent validation dataset.

### Statistical analyses

All analyses were performed in R software unless otherwise specified. The Wilcoxon rank-sum test and ANOVA were used for methylation level comparisons between two or more groups. Single-factor logistic regression was used to analyse the diagnostic power of each marker.

### Study approval

The collection of all samples was approved by ethics committees at Harbin Medical University Cancer Hospital, and all participants provided written informed consent prior to inclusion in the study.

### Reporting summary

Further information on research design is available in the Nature Research Reporting Summary linked to this article.

## Supplementary information

Supplementary Information

Dataset 1

Reporting Summary

## Data Availability

The sequencing data have been deposited at the European Genome-phenome Archive (EGA), which is hosted by the European Bioinformatics Institute, under study accession number EGAS00001004302. All other relevant data are available within the article or Supplemental Information or available from the authors on reasonable request.

## Code availability

R scripts are available upon request.
