# Supplementary material for: Circulating cell-free DNA-based methylation patterns for breast cancer diagnosis
Source: NPJ Breast Cancer. 2021 Aug 16;7:106. doi: 10.1038/s41523-021-00316-7 (PMC8367945; doi:10.1038/s41523-021-00316-7)
Supplement: Supplementary file 1 — Supplementary Information [file 41523_2021_316_MOESM1_ESM.pdf]

Supplementary Materials

Supplementary Figures &Tables

Supplementary Data 1：

Supplementary Table 3. Clinicopathological information for each breast cancer patient (*n*=204).

Supplementary Table 4. Methylation level of 26 markers in all plasma samples (*n*=333).

a

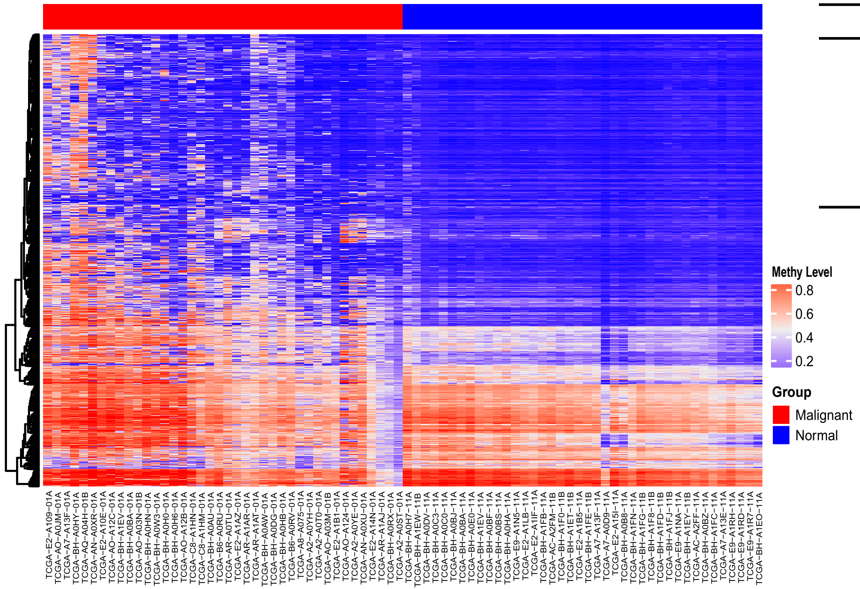

b

Gene types	count	percent
all	3288	100%
promoter	1658	50.43%
body	976	29.65%
intergenic	599	29.68%
3UTR	55	1.67%

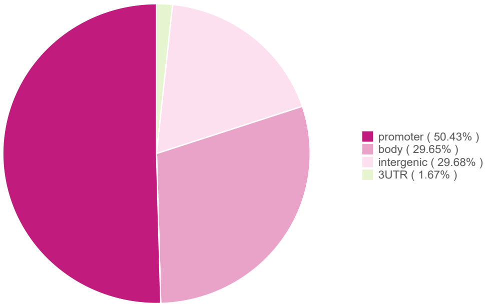

Supplementary Figure 1. Candidate methylation panel from TCGA data analysis. (**a**), Heatmap of the methylation level of 3,288 CpG sites in breast cancer based on TCGA data. (**b**), Distribution of targeted CpG sites relative to CpG islands and gene feature categories.

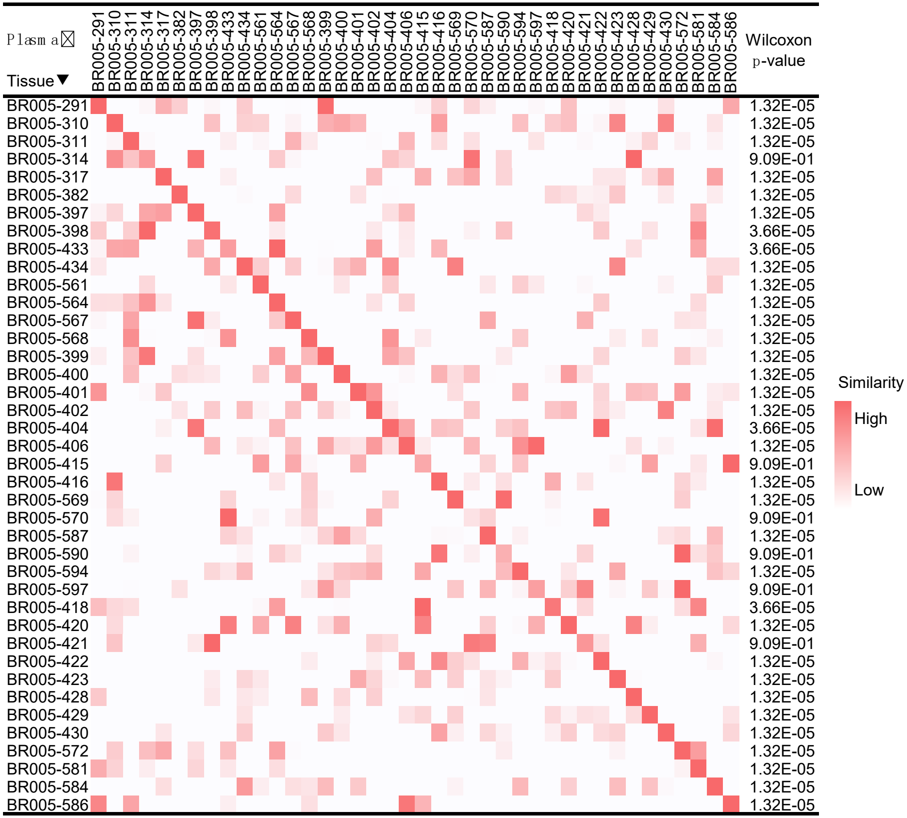

Supplementary Figure 2. High concordance of specific methylation patterns in matched tumour tissue and plasma samples. Read-level similarity of methylation patterns between matched tissue and plasma samples was calculated as the percentage of reads sharing predefined methylation patterns for a pair of tissue (row) and plasma (column) samples. Specifically, the similarity of a tissue sample to its matched plasma is shown in the diagonal of the heatmap, with the rank and Wilcoxon test p-value of the pair compared to the rest of the plasma samples shown on the right side of the heat map.

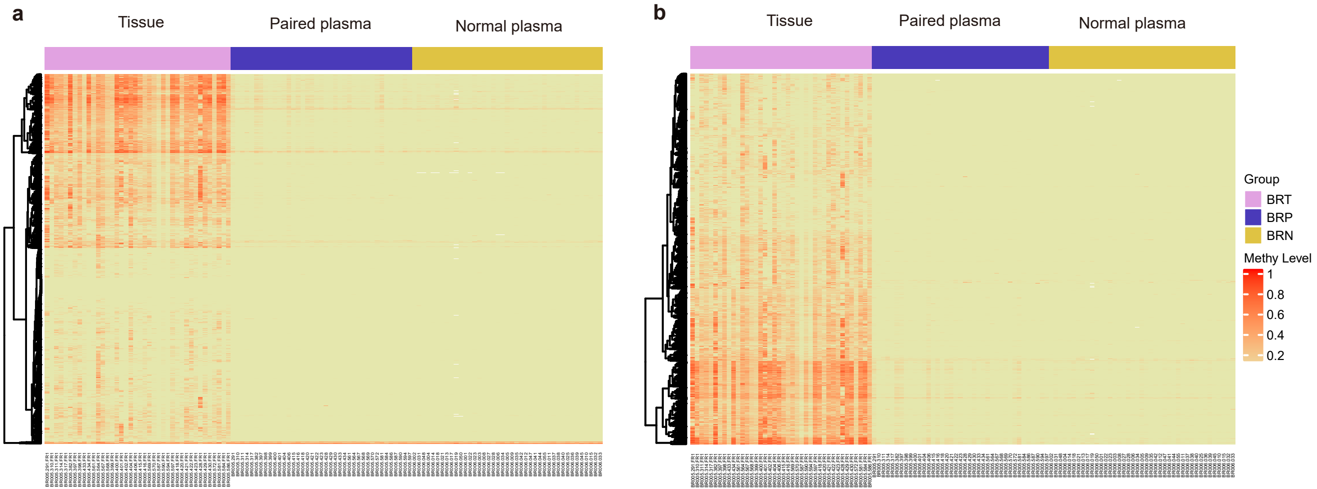

Supplementary Figure 3. Unsupervised hierarchical clustering of 3,288 and 1,996 differentially methylated markers between breast cancer tissue DNA and cfDNA. Heatmap of the methylation levels of 3,288 CpG sites (**a**) and 1,996 CpG sites (**b**) in normal plasma, breast cancer tumour tissues and paired plasma. Each column represents an individual patient, and each row represents a CpG marker. The scale represents the methylation beta values.

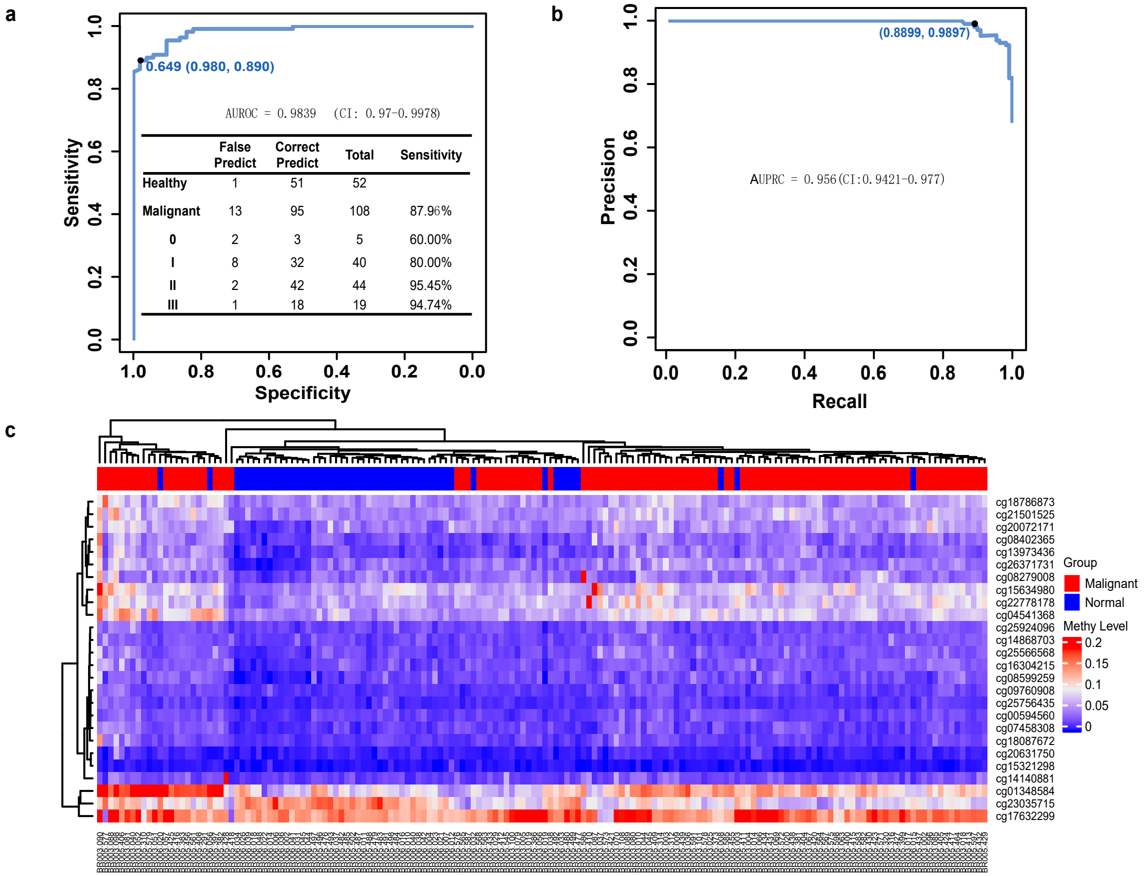

Supplementary Figure 4. ROC and PRC curve for breast cancer detection and heatmap of the cfDNA methylation levels in the training dataset using the final model with 26 markers. This model achieved the best diagnostic power with an AUROC of 0.9839 (95% CI: 97.0-99.78%) (**a**) and AURPC of 0.956 (95% CI: 94.21-97%) (**b**) and high sensitivity and specificity (**a**) in the training cohorts. Cancer or normal individuals can be distinguished through the methylation pattern of cfDNA in the training dataset (**c**).

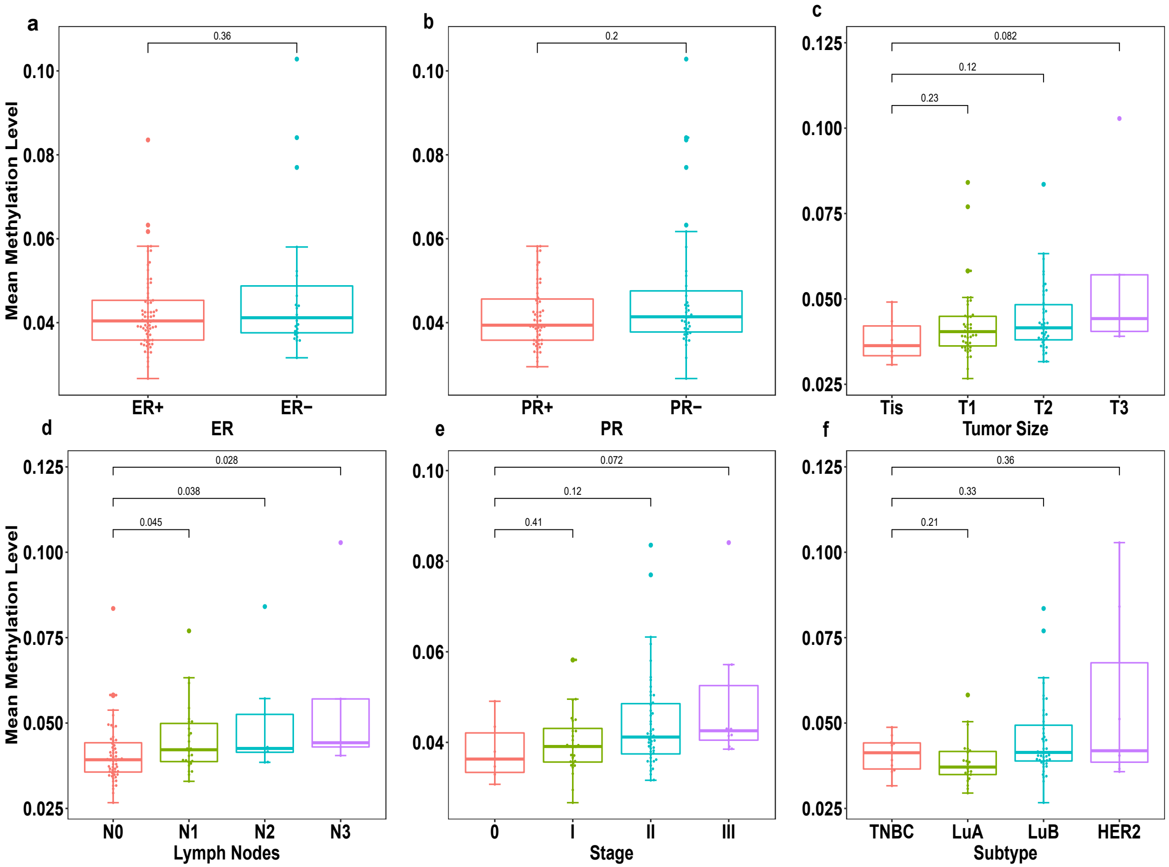

Supplementary Figure 5. Box plot comparing ER status (**a**), PR status (**b**), tumour size (**c**), number of metastatic lymph nodes(**d**), T stage (**e**) and breast cancer subtype (**f**) according to hypermethylation levels. For each box plot, the centre line, the boundaries of the box, the ends of the whiskers and points beyond the whiskers represent the median value, the interquartile range, the minimum and maximum values, and the outliers, respectively. For each box plot, the centre line, the boundaries of the box, the ends of the whiskers and points beyond the whiskers represent the median value, the interquartile range, the minimum and maximum values, and the outliers, respectively.

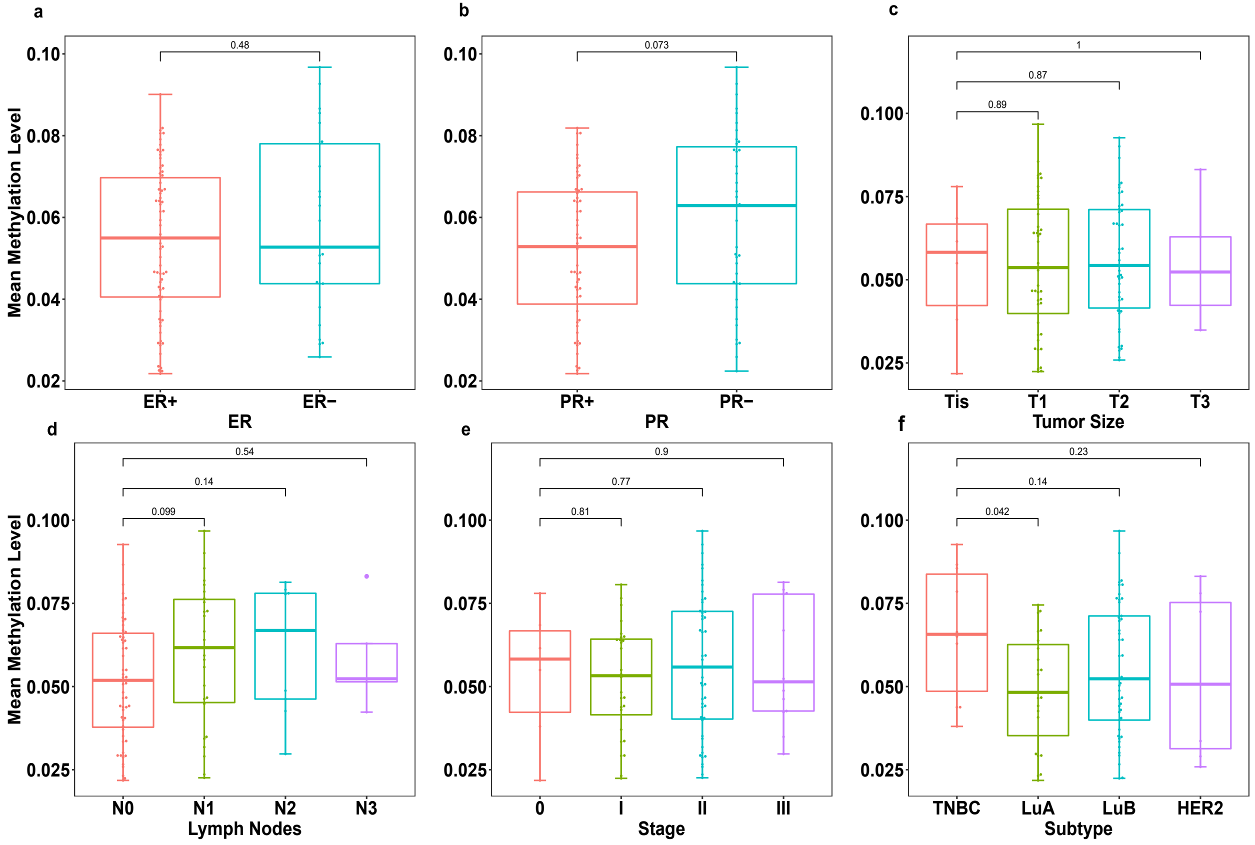

Supplementary Figure 6. Box plot comparing the ER status (**a**), PR status (**b**), tumour size (**c**), number of metastatic lymph nodes (**d**), T stage (**e**) and breast cancer subtype (**f**) according to hypomethylation levels. For each box plot, the centre line, the boundaries of the box, the ends of the whiskers and points beyond the whiskers represent the median value, the interquartile range, the minimum and maximum values, and the outliers, respectively. For each box plot, the centre line, the boundaries of the box, the ends of the whiskers and points beyond the whiskers represent the median value, the interquartile range, the minimum and maximum values, and the outliers, respectively.

Supplementary Table 1. The diagnostic power of each single differentially methylated region in breast cancer.

Region	Ref Gene	Coefficient[A]	*P* value	*P* adjust	Specificity	Sensitivity	AUC
cg23035715	*TLR5*	-99.22	1.63E-19	4.24E-18	0.89	0.90	0.9395
cg26371731	*C1orf61*	62.65	2.32E-07	6.03E-06	0.79	0.71	0.8020
cg04541368	*NR2F1-AS1*	44.73	3.03E-08	7.88E-07	0.81	0.70	0.7701
cg13973436	*ZDHHC1*	70.14	2.55E-08	6.63E-07	0.68	0.76	0.7511
cg16304215	*OTP*	90.15	1.24E-06	3.22E-05	0.76	0.70	0.7492
cg20072171	*FEZF2*	60.62	2.17E-07	5.64E-06	0.81	0.64	0.7478
cg08402365	*METAP1D*	59.46	1.22E-06	3.17E-05	0.70	0.75	0.7337
cg21501525	*TSHZ3, THEG5*	68.01	1.24E-07	3.22E-06	0.64	0.80	0.7334
cg22778178	*FLJ45513, DLX4*	50.98	1.66E-06	4.32E-05	0.78	0.65	0.7259
cg08599259	*POU3F1*	85.09	1.18E-06	3.07E-05	0.73	0.73	0.7213
cg25566568	*NKX2-1-AS1, NKX2-8*	104.18	9.18E-09	2.39E-07	0.81	0.61	0.7061
cg15634980	*PNPLA1*	40.20	1.08E-06	2.81E-05	0.63	0.74	0.7018
cg07458308	*TIFAB, NEUROG1*	96.18	8.80E-06	2.29E-04	0.70	0.64	0.6902
cg01348584	*RADIL*	18.72	3.33E-05	8.66E-04	0.67	0.70	0.6886
cg14140881	*EFCAB10*	55.80	9.23E-04	2.40E-02	0.70	0.66	0.6823
cg25756435	*MAST1*	104.57	3.41E-06	8.87E-05	0.68	0.66	0.6755
cg00594560	*ZFHX4-AS1*	140.50	4.75E-06	1.24E-04	0.76	0.57	0.6707
cg08279008	*POLR1A*	41.53	2.93E-05	7.62E-04	0.71	0.63	0.6644
cg09760908	*CAMKMT, SIX3-AS1*	117.72	6.42E-05	1.67E-03	0.57	0.78	0.6599
cg18087672	*HOXB13, TTLL6*	112.70	3.24E-06	8.42E-05	0.65	0.70	0.6588
cg14868703	*LHX5-AS1*	113.97	1.22E-05	3.17E-04	0.73	0.64	0.6460
cg17632299	*LECT1*	14.61	1.38E-03	0.036	0.60	0.73	0.6443
cg18786873	*ALX3*	49.94	4.55E-05	1.18E-03	0.59	0.71	0.6415
cg20631750	*BMP7*	178.72	2.23E-04	5.80E-03	0.67	0.65	0.6398
cg25924096	*PRDM13*	84.91	9.38E-04	0.024	0.38	0.86	0.6030
cg15321298	*ULBP1, RAET1K*	-79.54	5.17E-01	1.00	0.54	0.64	0.5399

[A]Regression coefficient in logistic regression models. Coefficient=ln (OR), OR: Odds ratio.

Supplementary Table 2. Associations between cfDNA methylation levels (26 markers) and clinical parameters (n=204).

Factors	Patients Num (n=204)	Hyper Methylation level (mean ± SD)	P value[A]	P adjust	Hypo Methylation level (mean ± SD)	P value[A]	P adjust
Age							
< 50	91	0.0430±0.0010	0.18	1.00	0.0452±0.0015	0.056	1.00
≥50	113	0.0455±0.0011			0.0502±0.0017		
Grade							
2	91	0.0436±0.0009	0.91	1.00	0.0480±0.0017	0.72	1.00
3	54	0.0447±0.0015			0.0498±0.0026		
ER							
+	148	0.0431±0.0007	0.12	1.00	0.0469±0.0013	0.22	1.00
-	50	0.0480±0.0022			0.0510±0.0027		
PR							
+	130	0.0429±0.0007	0.16	1.00	0.0456±0.0013	0.026*	0.494
-	65	0.0472±0.0018			0.0524±0.0024		
Her2							
+	47	0.0464±0.0023	0.61	1.00	0.0496±0.0026	0.38	1.00
-	135	0.0446±0.0012			0.0474±0.0016		
T							
Tis	11	0.0399±0.0019	5.9E-08*	1.12E-06*	0.0487±0.0051	0.42	1.00
T1	99	0.0426±0.0009			0.0465±0.0017		
T2	85	0.0449±0.0010			0.0488±0.0018		
T3	9	0.0641±0.0080			0.0556±0.0045		
N							
N0	123	0.0422±0.0007	1.2E-05*	2.3E-04*	0.0459±0.0014	0.18	1.00
N1	49	0.0451±0.0015			0.0515±0.0027		
N2	18	0.0474±0.0027			0.0506±0.0043		
N3	14	0.0568±0.0057			0.0508±0.0040		
Stage							
0	11	0.0399±0.0019	7.3E-05*	1.39E-03	0.0487±0.0051	0.28	1.00
I	68	0.0411±0.0009			0.0446±0.0018		
II	91	0.0446±0.0010			0.0496±0.0019		
III	32	0.0506±0.0026			0.0490±0.0028		
Subtype							
Luminal A	53	0.0413±0.0011	0.029*	0.55	0.0450±0.0019	0.18	1.00
Luminal B (HER2-)	58	0.0455±0.0013			0.0463±0.0023		
Luminal B (HER2+)	31	0.0443±0.0018			0.0509±0.0032		
HER2	15	0.0517±0.0061			0.0457±0.0048		
TNBC	23	0.0446±0.0021			0.0541±0.0040		

Grade, Histological grading of breast cancer. ER, estrogen receptor. PR, progesterone receptor. HER2, human epidermal growth factor receptor 2. [A]Wilcoxon rank-sum test or analysis of variance (ANOVA).* P value < 0.05 is statistically significant
