# Supplementary material for: Circulating cell-free DNA-based methylation patterns for breast cancer diagnosis
Source: NPJ Breast Cancer. 2021 Aug 16;7:106. doi: 10.1038/s41523-021-00316-7 (PMC8367945; doi:10.1038/s41523-021-00316-7)
Supplement: Supplementary file 3 — Reporting Summary [file 41523_2021_316_MOESM3_ESM.pdf]

nature portfolio

Corresponding author(s): Jian-Bing Fan,Da Pang

Last updated by author(s): Jul 17, 2021

Reporting Summary

Nature Portfolio wishes to improve the reproducibility of the work that we publish. This form provides structure for consistency and transparency in reporting. For further information on Nature Portfolio policies, see our Editorial Policies and the Editorial Policy Checklist.

Statistics

For all statistical analyses, confirm that the following items are present in the figure legend, table legend, main text, or Methods section.

n/a	Confirmed	
☐	☒	The exact sample size (*n*) for each experimental group/condition, given as a discrete number and unit of measurement
☒	☐	A statement on whether measurements were taken from distinct samples or whether the same sample was measured repeatedly
☐	☒	The statistical test(s) used AND whether they are one- or two-sided<br>*Only common tests should be described solely by name; describe more complex techniques in the Methods section.*
☐	☒	A description of all covariates tested
☐	☒	A description of any assumptions or corrections, such as tests of normality and adjustment for multiple comparisons
☐	☒	A full description of the statistical parameters including central tendency (e.g. means) or other basic estimates (e.g. regression coefficient) AND variation (e.g. standard deviation) or associated estimates of uncertainty (e.g. confidence intervals)
☐	☒	For null hypothesis testing, the test statistic (e.g. *F*, *t*, *r*) with confidence intervals, effect sizes, degrees of freedom and *P* value noted<br>*Give P values as exact values whenever suitable.*
☒	☐	For Bayesian analysis, information on the choice of priors and Markov chain Monte Carlo settings
☐	☒	For hierarchical and complex designs, identification of the appropriate level for tests and full reporting of outcomes
☐	☒	Estimates of effect sizes (e.g. Cohen's *d*, Pearson's *r*), indicating how they were calculated

Our web collection on statistics for biologists contains articles on many of the points above.

Software and code

Policy information about availability of computer code

Data collection	We used the GD C-Client(version"1.3.0") tool to download TCGA-BRCA data and pathological information of 40 paired breast cancer tissues and adjacent normal tissues. Then, designed probes corresponding to the 3,288 markers and tested them in 40 paired breast cancer tissue DNA and matched plasma cfDNA. Last,We obtained the targeted bisulfite sequencing of 336 cfDNA samples for further analysis.
Data analysis	All data were analysised on the R softeware(Version "3.3.3").At first,We use he LIMMA Package(version"3.30.13") to find the differential methylation markers through the 40 paired breast cancer tissues and adjacent normal tissues. Then,We designed the probes of the selected 3288 markers and obtain the quality control qualified targeted bisulfite sequences of 229 samples .Next we used sampling Package(version "2.8") to split sample into train set and validation set in 7:3 ratio .Then We narrowed the methylation markers to 26 markers by random forest (version "4.6.14")and LASSO(version "2.0.16") methods,tesed the classification effect of the model in validation set by random forest (version "4.6.14").Last,we obtained the targeted bisulfite sequences of 103 samples as the validation set to confirm the classification effect of model.

For manuscripts utilizing custom algorithms or software that are central to the research but not yet described in published literature, software must be made available to editors and reviewers. We strongly encourage code deposition in a community repository (e.g. GitHub). See the Nature Portfolio guidelines for submitting code & software for further information.

Data

Policy information about availability of data

All manuscripts must include a data availability statement. This statement should provide the following information, where applicable:

- Accession codes, unique identifiers, or web links for publicly available datasets
- A description of any restrictions on data availability
- For clinical datasets or third party data, please ensure that the statement adheres to our policy

The sequencing data have been deposited at the European Genome-phenome Archive (EGA), which is hosted by the European Bioinformatics Institute, under study accession number EGAS00001004302. All other relevant data are available within the article or Supplemental Information or available from the authors on reasonable request.

Field-specific reporting

Please select the one below that is the best fit for your research. If you are not sure, read the appropriate sections before making your selection.

☒ Life sciences ☐ Behavioural & social sciences ☐ Ecological, evolutionary & environmental sciences

For a reference copy of the document with all sections, see nature.com/documents/nr-reporting-summary-flat.pdf

Life sciences study design

All studies must disclose on these points even when the disclosure is negative.

Sample size	The prevalence of breast cancer in training and validation sets was 67%. The specificity of the diagnostic model was set at 90%, the confidence interval was set at 0.05, and the total number of samples calculated by the formula[(Zα/2^2*SP*(1-SP)/(L^2*(1-Prev)] is 206, including at least 138 cases of breast cancer patients and at least 68 cases of normal.
Data exclusions	Exclusioned[Incomplete clinical information (n=2) ,No pathological information (n=7),Failed sequencing QC(n=3)], and the exclusion criteria is pre-established.
Replication	AnchorIRIS™ assay library preparation and sequencing:Extracted cfDNA was bisulfite treated and purified using an EZ DNA Methylation-Lightning Kit (Cat# D5031, Zymo Research) according to the manufacturer's protocol. Whole-genome or cfDNA amplification of bisulfite-converted DNA was performed using an AnchorDx EpiVisioTM Methylation Library Prep Kit (AnchorDx, Cat# A0UX00019) and an AnchorDx EpiVisioTM Indexing PCR Kit (AnchorDx, Cat# A2DX00025) following recommended conditions. Target enrichment was performed using an AnchorDx EpiVisioTM Target Enrichment Kit (AnchorDx, Cat# A0UX00031) to specifically pull down DNA fragments that contained target CpG sites using 5'-biotinylated capture probes. A total of 1,000 ng DNA containing up to 4 pre-libraries was pooled for target enrichment using an AnchorDx PanMet V2-Pan-cancer methylation panel (AnchorDx, Cat# A0UX00023). Target capture libraries were sequenced on an Illumina NovaSeq 6000 Sequencer using 300 cycle runs. A 25% PhiX solution was spiked into the bisulfite sequencing libraries to increase base diversity for better sequencing quality.
Randomization	The train and validation data are split 7:3 and balanced with age, stage, and subtypes.The test set samples is subsequent collect.
Blinding	We chose between 40 to 60 years old of breast cancer patients and healthy people for enrolling, and try to ensure that all molecular subtypes are included in each breast cancer stage ,who clinical information is not complete or no pathological information are exclusioner. Then, all samples are subject to strict quality control (extract the purity of DNA sequencing, DNA concentration, coverage,Sequencing depth) in the experiment and sequencing, who with failed sequencing QC will be excluded. At the beginning, enrolling samples were 155 Malignant and 74 Normal. In these samples, we performed random segmentation in a 7:3 ratio, and tried to balance the ages, subtypes and stages of the two sets during the segmentation. For further modeling verification, 49 Malignant and 55 Normal samples were included under the same conditions.

Reporting for specific materials, systems and methods

We require information from authors about some types of materials, experimental systems and methods used in many studies. Here, indicate whether each material, system or method listed is relevant to your study. If you are not sure if a list item applies to your research, read the appropriate section before selecting a response.

Materials & experimental systems

n/a	Involved in the study
☒	☐ Antibodies
☒	☐ Eukaryotic cell lines
☒	☐ Palaeontology and archaeology
☒	☐ Animals and other organisms
☐	☒ Human research participants
☐	☒ Clinical data
☒	☐ Dual use research of concern

Methods

n/a	Involved in the study
☒	☐ ChIP-seq
☒	☐ Flow cytometry
☒	☐ MRI-based neuroimaging

Human research participants

Policy information about studies involving human research participants

Population characteristics	Participants included 204 female patients 37-76 years old, with an average age of 51 years and with histologically confirmed BC. Among these patients, 108 were in the training group, 47 were in the validation group, and 49 were in an independent testing dataset. A total of 129 healthy adult volunteers without BC participated in the study. Among these volunteers, 52 were in the training group, 22 were in the validation group, and 55 were in the independent testing group. The age distribution of these healthy donors was 40-60 years, with an average age of 49 years.
Recruitment	Female subjects 37-76 years old,with histologically confirmed BC and no other tumor was found in the medical history were enrolled into the study.A total of 129 healthy adult volunteers without BC participated in the study.All subjects must be informed of the investigational nature of this study and be willing to provide written informed consent in accordance with Institutional guidelines.
Ethics oversight	The collection of all samples was approved by ethics committees at Harbin Medical University Cancer Hospital.

Note that full information on the approval of the study protocol must also be provided in the manuscript.

Clinical data

Policy information about clinical studies

All manuscripts should comply with the ICMJE guidelines for publication of clinical research and a completed CONSORT checklist must be included with all submissions.

Clinical trial registration	Not applicable
Study protocol	This is a retrospective study. All details are provided in the Methods section
Data collection	Clinical data were retrospectively obtained from the database of Harbin Medical University Cancer Hospital.
Outcomes	The outcome measures were defined and pre-specified - as described in the manuscript.
